# Case of Small-pox after Vaccination

**Published:** 1813-07

**Authors:** Richard Henry King

**Affiliations:** Mortlake


*For the Medical and Physical Journal.*

To J. WALKER, M. D.

DEAR SIR,

I TAKE the liberty of sending you the inclosed case of Small-pox occurring after Vaccination, in a boy whom you vaccinated about nine years ago, in Salisbury-square. It may be necessary to state that the mother, who then lived at Brentford, never brought this child to you after the first day, he being then an infant in arms. His brothers and sisters, who were vaccinated about the same time, were regularly seen by you during the progress of the disease; and the mother assures me that in this patient the arm rose and went through the different stages as regularly as the others had done, and the cicatrix, although small, is now sufficiently visible. To this statement, however, I should have paid but little attention, (and the circumstance of his not having been seen by any medical man after the period of inoculation, made me consider it as a case of spurious or imperfect vaccination, and prevented my sending you earlier notice of it, or showing it to any other medical men,) but the speedy and favorable termination of the disease has now convinced me that the previous vaccination had assisted its beneficial effects on the constitution, and, although it had not entirely secured it from small-pox infection, had yet the power of arresting this disease at the moment when it had assumed the most formidable character, and rendering it harmless; and in this opinion I am happy to say that Mr. Andrews, a neighbouring practitioner, whom I this day requested to visit the subject of this communication, concurs with me. If you think the case is worthy of being laid before the public, as another proof of the prophylactic power of cow-pox, you are at full liberty to send it, together with any remarks of your own, to the Editors of the Medical and Physical Journal. I remain, dear Sir,

Your obedient humble Servant,

*Mortlake, May* 2, 1813. RICHARD HENRY KING.

About three weeks or a month ago, a girl in the poor-house of the parish of Mortlake, who was supposed to have had the small-pox in her infancy, but concerning which I regret my not having been able to obtain any satisfactory particulars, sickened with that disease, and had it in a very favorable manner. As all the other inmates of the house had had either the small-pox or the cow-pox, I did not think it necessary to use any particular precautions in keeping this patient separate from them. On the 23d of April, however, Thomas Waite, aged about nine years, who had been vaccinated in Salisbury-square when six weeks old, was seized with rigor and shivering, followed by head-ache, pain in his back, and nausea. When I saw him, his pulse was very quick, and the tongue was covered with a brown fur. 24th. The fever had increased; he had passed a restless night, had been slightly delirious, and still complained of sickness at his stomach. It may be necessary to state, that he had taken an emetic the preceding evening, and a purgative medicine this morning, both of which had operated sufficiently. 25th. He had been delirious during the whole night, and his fever continued very high. In the course of this day an eruption appeared all over him, resembling measles. 26th. From the appearance of the eruption this morning, there was now little doubt of its being small-pox of the most unfavorable kind. He had again been delirious during the night, and the fever had not at all decreased, but the vomiting had now subsided. From this period till the 29th, the disease continued its progress, daily assuming a more unfavorable aspect. The eruption consisted of minute, but very numerous vesicles, which, on the face, already showed a disposition to run into each other. 30th. On this morning, being the sixth day of the eruption, the constitutional symptoms had undergone a most favorable change. The fever had abated, the boy had passed a quiet night, and he expressed some inclination for food, which he had not previously done. The appearance of the eruption, however, was still by no means flattering. The vesicles continued small and flat: in the interstices between them, the skin was pale and shrunk, and there was not the least tendency to swelling in the face and eyes. Still, however, the general appearances appearances were such as led me to hope that the previous vaccination would exert a favorable influence, so as to arrest the further progress of this apparently alarming disease; and this opinion I did not hesitate to express to the attendants. May 1st. On this day my hopes were confirmed. Almost all the vesicles had dried up and turned brown in the course of the night; the boy had slept well, was perfectly free from fever, and his appetite for food had returned. From this time he continued to mend rapidly, and he is now (May 5th) as nearly well as possible. On his face there is now very little appearance of the eruption remaining. I have not thought it necessary to give a regular detail of the treatment of this case during the progress of the disease. At the commencement, his stomach and bowels were cleared, the antiphlogistic regimen was adhered to during the eruptive fever, and for the last three or four days he had taken bark and wine very freely. About the fourth day of the eruption he was attacked with diarrhœa, and voided several copious, dark-colored, and very offensive stools.

This boy has three or four brothers and sisters in the house, who were vaccinated at the same time with himself, and who, as well as all the other children, have hitherto escaped infection, unless, indeed, a slight indisposition of his elder brother, William Waite, can be attributed to this cause. The mistress brought this boy to me on the 1st of May. He had been drooping for a day or two, complained of pain in his head and back, and had vomited once or twice. His whole body was now covered with an efflorescence exactly resembling the first appearance of the eruption in his brother Thomas; but this entirely disappeared on the following day, and he has continued perfectly well ever since. What is remarkable in this case is, that the boy complained of a severe pain on the spot where the vaccine matter had been inserted, and said he had felt it during the whole of the day preceding that on which I first saw him; but this also ceased with the disappearance of the efflorescence.

From the girl who first had the small-pox, I was induced to inoculate a child whom I had previously vaccinated, but of whose security I had some doubts, owing to the vesicle being ruptured previous to the appearance of the areola. The arm inflamed and maturated, but there was no constitutional affection.

May 6th.—Since writing the inclosed, I have been informed by the mistress of the house that Elizabeth Waite was affected in a similar manner with her brother William, about the time that Thomas Waite first began to complain. She

 complained complained for a couple of days of languor and loss of appetite. On the third day a rash appeared all over her, continued for two days, and then disappeared. As her indisposition was extremely slight, it was unfortunately not mentioned to me.

